# The Relationship Between Alexithymia and Mobile Phone Addiction Among Mainland Chinese Students: A Meta-Analysis

**DOI:** 10.3389/fpsyt.2022.754542

**Published:** 2022-02-10

**Authors:** Haitao Huang, Xiao Wan, Guangli Lu, Yueming Ding, Chaoran Chen

**Affiliations:** ^1^Institute of Nursing and Health, Henan University, Kaifeng, China; ^2^School of Business, Henan University, Kaifeng, China

**Keywords:** alexithymia, mobile phone addiction, mainland Chinese students, meta- analysis, review

## Abstract

Alexithymia and mobile phone addiction are common phenomena in daily life. Many studies have explored the internal relationship between them based on different theoretical perspectives, but the extent of the exact correlation is still controversial. To address this controversy and clarify the reasons for the divergence, a meta-analysis of 26 articles comprising 23,387 Chinese students was conducted. The results show that alexithymia was highly positively correlated with mobile phone addiction (r = 0.41, 95% CI = [0.37, 0.45]). Furthermore, the relationship was moderated by mobile phone addiction measurement tool and year of publication, with studies using the Mobile Phone Addiction Tendency Scale (MPATS) having higher correlation coefficients than those using the Mobile Phone Addiction Index (MPAI) or other measurement tools. Studies published in 2020–2021 yielded higher correlations than those published in 2014–2016 and 2017–2019. However, the relationship was not moderated by gender, region, or measures of alexithymia. Therefore, our meta-analysis of available published data indicated that alexithymia and mobile phone addiction in Chinese students are not only highly positively correlated but also affected by mobile phone addiction measurement tools and publication year. Longitudinal studies or experimental studies should be strengthened in the future to further establish the direction(s) of causality for the relation between alexithymia and mobile phone addiction.

## Introduction

Globally, mobile phones have become an indispensable part of daily life. Smartphones can perform a variety of functions, such as internet surfing, e-mail management, online games and social networking (1, 2). Over the past decade, smartphone ownership has soared, especially among young people in Europe and Asia. According to the “The 47th China Statistical Report on Internet Development,” as of December 2020, the number of mobile internet users of teenagers aged 10–19 has reached 136 million, accounting for 13.5% of the total number of internet users. In different occupational compositions, the proportion of students is the highest, at 21.0% (3).

Although smartphones can help teenagers with online communication, shopping, entertainment, learning and other activities and bring great convenience to their study and life, it should be noted that an increasing number of teenagers are finding it difficult to eliminate the use of mobile phones, which leads to the emergence of mobile phone addiction (MPA) (4, 5). MPA, also known as mobile phone dependence and problematic mobile phone use, refers to the psychological dependence caused by the excessive use of smartphones, which leads to the loss of control over the use of smartphones and related services, resulting in the interference of daily life and psychological or behavioral problems (6, 7). Studies have shown that MPA can lead to headaches, earaches, changes in brain structure (8), reduced life satisfaction (9), blurred self-concept (10), reduced academic achievement (9) and other mental health problems, such as anxiety and depression (11). Unfortunately, it was reported that students were more vulnerable to MPA (12). A survey of Chinese students' MPA shows that compared to older social groups, Chinese students are usually mentally immature and have less self-regulatory ability (13). Therefore, they are more likely to use mobile phones excessively. The prevalence of MPA among students was 21.3% in China (12); for comparison, the prevalence was 20% in Spain (14), 18.5% in India (15), 10% in British (16). In addition, a recent survey of Chinese students showed that Chinese students' MPA was weakly to moderately positively correlated with anxiety, depression, impulse and sleep quality (17). Therefore, this paper takes students in mainland China as the research object to discuss the problems related to MPA, which is of great significance to the physical and mental health of students in mainland China.

Previous researchers have investigated whether self-esteem (18), loneliness (19) or lifestyle (20) are linked to MPA. As a special personality trait, the relationship between alexithymia and Chinese students' MPA has also been widely investigated (21–23). Used for the first time by Sifneos to describe certain clinical characteristics observed among psychosomatic patients, the term alexithymia refers to a multidimensional personality construct, defined by a set of four characteristics: 1) difficulty in identifying feelings and in distinguishing feelings from bodily sensations of emotional arousal, 2) difficulty in describing and in communicating feelings to others, 3) lack of fantasy and imagination, and 4) an externally oriented style of thinking (24, 25). Studies have found that alexithymia is closely related to the occurrence of negative emotions. First, adolescents with different alexithymia levels showed significant differences in depressive symptoms, with adolescents showing more depressive emotions as alexithymia symptoms increased (26). Second, alexithymia is an important predictor of anxiety, and a reduction in alexithymia can alleviate anxiety symptoms (27). In addition, there was also a significant positive correlation between alexithymia and stress. The alexithymia stress hypothesis posits that individuals with alexithymia characteristics make negative and exaggerated assessments of their environment due to inappropriate descriptions of their emotions, thus affecting their assessment of challenges and threats and ultimately putting themselves in a stressful state (28). Further studies have shown that alexithymia is a risk factor for increasing anxiety, depression and stress, which can eventually lead to mental illness (29). and even suicide risk (30, 31). The self-medication model of drug use disorder suggests that adolescents with negative emotions may improve their emotional status by using the internet or sending text messages, because it is considered to be less harmful than illegal drugs and easier to obtain (32). Finally, alexithymia plays an essential role in the etiopathogenesis of addictive disorders. For example, alexithymia has a significant positive correlation with the severity of alcohol addiction (33), eating disorders (34) and pathological gambling (35). Stratified regression analysis showed that alexithymia was an important predictor of Internet addiction (36). Another study showed that the alexithymia score of potential mobile phone addicts was significantly higher than that of the control group, and it was difficult for potential mobile phone addicts to control their mobile phone use (37). Therefore, alexithymia may not only directly affect MPA but also indirectly affect MPA in mainland Chinese students through mediating factors such as anxiety and depression.

Currently, the screening instruments for MPA mainly include the Mobile Phone Addiction Index (MPAI) (38), the Mobile Phone Addiction Tendency Scale for College Students (MPATS) (39), the Smartphone Addiction Inventory (SPAI) and its Brazilian version (SPAI-BR) (40) and the Smartphone Addiction Scale (SAS) and its short version (SAS-SV) (41). In China, the most commonly used scales for MPA are the MPAI and MPATS. The MPAI consists of 17 items and is rated on a Likert-type scale ranging from 1 (“almost none”) to 5 (“always”), including four dimensions of loss of control, abstinence, avoidance and inefficiency. Higher scores indicate a more severe phone addiction. The MPATS mainly includes four dimensions: withdrawal symptoms, prominent behavior, social comfort and emotional change. The scale has 16 items and adopts a Likert 4-point system. A higher score indicates a more severe level of MPA. Similarly, the most commonly used questionnaires for alexithymia in China are the Toronto Alexithymia Scale-20 (TAS-20) (42) Alexithymia Questionnaire of College Students (AQCS) (43). The TAS-20 consists of 20 items with three factors: difficulty in identifying feelings, difficulty in describing feelings and externally oriented thinking. It was rated on a five-point scale, ranging from 1 (strongly disagree) to 5 (strongly agree), with a total score ranging from 20 to 100. All items were summed to create a composite score for each participant, with higher scores indicating higher levels of alexithymia. In traditional TAS-20 cutoffs, a total score >60 indicated that the participant had alexithymia (42). The AQCS consists of 23 questions across 4 dimensions: difficulty identifying feelings, difficulty describing feelings, difficulty analyzing feelings and difficulty experiencing feelings. This tool is rated on a 5-point rating scale with “1: very inconsistent, 5: very consistent.” Higher scores indicate higher levels of alexithymia.

Evidence thus far has shown that alexithymia is associated with MPA in Chinese students. However, the strength of identified associations has varied considerably thus far, ranging from small (r = 0.23) (22) to large (r = 0.57) (21). One of the reasons for this debate is the small sample size of individual studies. Meta-analysis can compile all past studies for a much larger effective sample size to determine an overall relation between alexithymia and MPA. Noting the lack of systemic meta-analyses examining these quantitative studies on the relationship between alexithymia and MPA among Chinese students. The current study was conducted to explore the relationship between alexithymia and MPA among Chinese students to provide evidence on the strength of the correlation between the two factors.

Moreover, the mixed results might stem from differences in the measures [of alexithymia or MPA (44)] or from demographic differences across the studies' participants (45). As most past studies did not account for moderators, this study also examines whether the relation between alexithymia and MPA differs across (a) the choice of alexithymia measure; (b) the choice of MPA measure; and (c) the demographic profile of the sample (age, gender, region).

### Purpose of This Study

This study aims to synthesize the results of previous studies concerning the relation between alexithymia and MPA among Chinese students and to identify some factors that influence this relationship. Specifically, this study (a) calculates an overall effect size for the relation between alexithymia and MPA and (b) tests whether the choice of measures or demographic variables moderates this relation. These objectives were addressed using a meta-analysis, which helps to identify the source of interstudy variability and can uncover interesting associations between studies.

## Methods

The study was designed and written according to the Preferred Reporting Items for Systematic Reviews and Meta-Analyses statement (46, 47).

### Literature Research

Six databases were searched for studies on the relationship between alexithymia and MPA: PubMed, Embase, Web of Science, China National Knowledge Infrastructure (CNKI), Wanfang data and Chongqing VIP Information Co., Ltd. (VIP). The retrieval time was from the establishment of the database to May 15, 2021. For English databases, the key words were (“Cell phone^*^ OR Cellular phone^*^ OR Cellular telephone^*^OR Mobile devices OR Mobile phone OR Smart phone OR Smartphone”) AND (“Addiction OR Dependence OR Dependency OR Abuse OR Addicted to OR Overuse OR Problem use OR Compensatory use”) OR (“Problematic smartphone use OR Problematic smartphone use OR Problematic mobile phone use OR Problematic cell phone use OR Nomophobia”) AND (“Alexithymia OR Affective Symptom OR Symptom, Affective OR Symptoms, Affective OR Alexithymia OR Alexithymia OR Emotional Disturbances OR Disturbance, Emotional OR Disturbances, Emotional OR Emotional Disturbance”). For Chinese databases, the key words all Chinese, namely (“Alexithymia”) AND (“Mobile phone addiction OR Problematic smartphone use”). A detailed search strategy is available in Supplementary Material 1. In addition, a gray literature search was performed using Google Scholar and CNKI to capture dissertations, theses that met the inclusion criteria. Publication languages were limited to English and Chinese. The reference lists of the retrieved articles were also manually checked to identify additional relevant papers.

### Study Selection Criteria

All literature records were independently screened against the following selection criteria by two reviewers for potentially eligible articles: (1) cross-sectional studies offering Pearson's correlation coefficients for the associations between MPA and alexithymia; (2) the article reports sample size; (3) the sample is mainly from Chinese mainland student, excluding prisoners or sick individuals; (4) when multiple publications use the same dataset, the dataset published in the academic journal is used, but if the journal article does not use the complete dataset, the original paper analyzing the complete dataset is used; (5) conference abstracts and review articles were excluded. The selection process included reading the title, abstract and full text of the article, and 26 articles ultimately met the inclusion criteria.

### Data Extraction

Data were independently extracted by two authors using a purpose-designed form. The following information was extracted: first author, year of publication, geographic location, literature type, sample size, instruments used to measure the degree of MPA, instruments used to measure alexithymia, and Pearson's correlation coefficients between MPA and alexithymia. Any disagreements were first discussed between these two authors and further disagreements were arbitrated by a third author.

### Quality Assessment

The methodological quality of the included cross-sectional studies was assessed using an 11-item checklist that was recommended by the Agency for Health care Research and Quality (AHRQ) (48). The 11 evaluation items are as follows: 1) define the source of information (survey, record review); 2) list inclusion and exclusion criteria for exposed and unexposed subjects (cases and controls) or refer to previous publications; 3) indicate time period used for identifying patients; 4) indicate whether or not subjects were consecutive if not population-based; 5) indicate if evaluators of subjective components of study were masked to other aspects of the status of the participants; 6) describe any assessments undertaken for quality assurance purposes (e.g., test/retest of primary outcome measurements); 7) explain any patient exclusions from analysis; 8)describe how confounding was assessed and/or controlled; 9) if applicable, explain how missing data were handled in the analysis; 10) summarize patient response rates and completeness of data collection; 11)clarify what follow-up, was expected and percentage of patients for which incomplete data or follow-up was obtained. The answer to each item was “no,” “unclear,” and “yes,” respectively. Study quality was defined as follows: low quality (0–3 yes), moderate quality (4–7 yes), and high quality (8–11 yes). The methodological quality of all studies included was independently assessed by two researchers. A third author was consulted to resolve any differences.

### Statistical Analysis

Effect sizes were calculated through the Pearson product-moment correlation coefficient (r). Since the variance depended strongly on the correlation, the r-coefficient was converted to Fisher's z scale. The transformation from the sample correlation r to Fisher's z is given by formula (1) and the standard error is calculated by formula (2), where n is the sample size. Fisher's z statistic is assumed for normally distributed data, and the 95% confidence interval was computed as formula (3) Finally, an inverse transformation was performed to report the results on the scale of the r-coefficient through formula (4).

(1) $Fisher's\;Z=0.5\;\chi \ln\frac{1+r}{1-r}$
(2) $S_{E}=\sqrt{1/\left(n-\;3\right)}$
(3) 95*%CI* = *Z*± 1.96(*SEz*)
(4) $Summary\;r=\frac{e^{2z}-1}{e^{2z}+1}$ (Z = summary Fisher's Z).

Considering that the heterogeneity of the included studies may affect the results, the random effects model was selected (49). The meta-analysis was performed with the Der-Simonian and Laird's method (50), where the weighting of sample size was introduced into the model as the inverse of variance.

To determine the heterogeneity of the effect sizes, we calculated both the Q statistic and *I*^2^. A Q statistic tests the hypothesis that the observed variance in effect sizes is no greater than that expected by sampling error alone, while *I*^2^ quantifies the dispersion. The *I*^2^ statistic may be interpreted (with caution) as follows: <25, 50, and >75% indicate low, moderate and large heterogeneity, respectively (51). For categorical variables, subgroup analyses were performed to identify potential factors, such as assessment tool, age group and region, which may influence the association between alexithymia and the MPA. Q_B_ was used to explore the impact of categorical variables on the effect size, and *P* < 0.05 was considered statistically significant. In addition, we also conducted a meta-regression analysis of the female ratio to examine whether gender influences the relationship between MPA and alexithymia in Chinese students. Since the reference standard for the interpretation of the correlation coefficient proposed by Cohen (52) (r = 0.1 is low correlation, r = 0.3 is medium correlation and r = 0.5 is strong correlation) is based on qualitative analysis, it is relatively subjective. Therefore, this paper adopts the suggestions of Gignac and Szodorai (53), and r = 0.1, r = 0.2 and r = 0.3 represent a low correlation, medium correlation and strong correlation respectively.

To evaluate the influence of individual studies on the summary correlation coefficients and test the robustness of the correlations between MPA and alexithymia, sensitivity analyses were conducted by sequentially omitting one study each turn. Visual inspection of funnel plots, Egger's linear regression test (54) and the trim-and-fill test (55) were performed to help us assess publication bias. All data were analyzed using Stata 16.0.

## Results

### Characteristics of Included Studies and Quality Assessment

**Figure 1:**
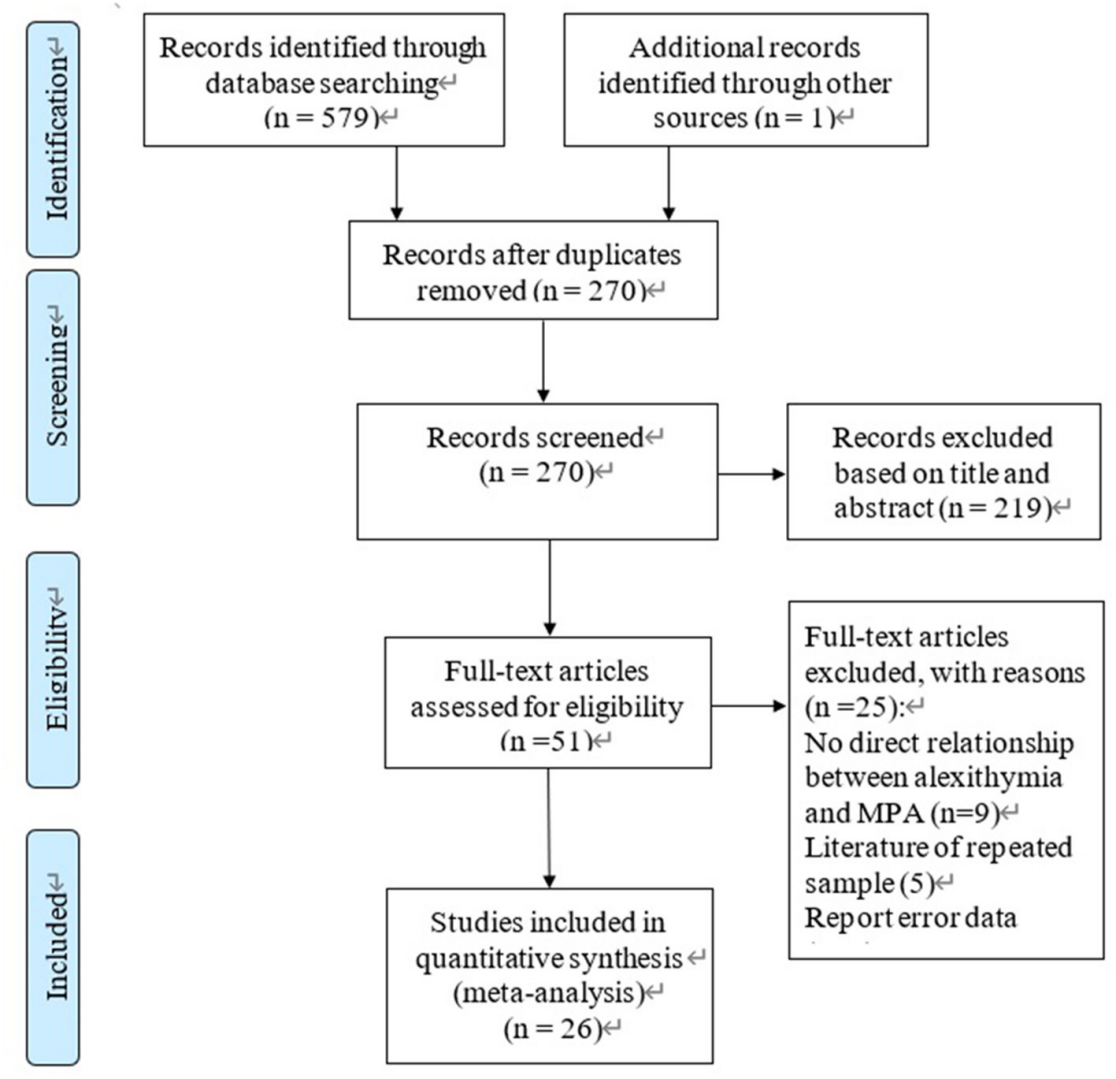
Flow chart of the study selection process.

The flow chart is shown in Figure 1. The literature search resulted in 580 studies with one study added after reviewing the gray literature. After eliminating the duplicates, 270 articles remained. There were 219 studies excluded according to titles and abstracts. Finally, the full texts of 51 articles were reviewed. We excluded 25 studies because they were either irrelevant, not correlation studies, unavailable full text, or had apparent data mistakes or reviews. As shown in Table 1, 26 studies ultimately met the inclusion criteria, involving a total of 23,387 participants. The sample sizes of the studies ranged from 220 to 4,147. The 11-item checklist recommended by the AHRQ was used to assess the papers. Seven studies were of high quality and 19 studies were of moderate quality (Supplementary Material 2).

**Table 1:** Characteristics of the 26 studies included in the meta-analysis.

**References**	**Region**	**Journal**	**Group**	***N***	**Female (%)**	***r***	**MPA scale**	**Alexithymia scale**
Wang (56)	Central	Dissertation	C	751	0.58	0.36	MPAI	TAS-20
Zhang (57)	Central	General	C	4,147	0.69	0.37	SQAPMPU	TAS-20
Zheng (58)	Central	General	M	742	0.43	0.54	MPAI	TAS-20
Li (59)	Central	Dissertation	C	1,105	0.52	0.33	MPAI	TAS-20
Hou et al. (60)	Central	General	C	611	0.37	0.43	MPAI	TAS-20
Chen (61)	Eastern	General	C	346	0.71	0.37	CSMPDQ	TAS-20
Wu (62)	*N*	General	C	220	0.55	0.41	CSMPDQ	TAS-20
Sun et al. (63)	Eastern	General	C	684	0.43	0.26	MPAI	TAS-20
Gao et al. (22)	Eastern	General	C	1,105	0.52	0.23	MPAI	TAS-20
Zhang (64)	Western	General	C	472	0.56	0.40	MPAI	TAS-20
Mei et al. (65)	Central	General	C	1,034	0.91	0.31	MPATS	TAS-20
Hao (66)	Eastern	Dissertation	M	1,447	0.41	0.30	MPAI	TAS-20
Xu (67)	Central	General	M	511	0.43	0.36	MPATS	AQCS
Huang et al. (68)	Central	General	C	479	0.65	0.48	MPAI	TAS-20
Chen and Shao (69)	Eastern	General	C	547	0.30	0.39	MPATS	TAS-20
Lin (70)	Eastern	Dissertation	M	453	0.47	0.56	MPATS	TAS-20
Li (71)	Central	General	M	693	0.46	0.38	MPAI	TAS-20
Hao et al. (23)	Eastern	General	C	847	0.49	0.34	MPAI	TAS-20
ARN (72)	Central	Dissertation	C	519	0.34	0.27	MPAI	TAS-20
Zhu (73)	Central	General	C	491	0.43	0.41	MPATS	AQCS
Huang and Zhao (74)	Central	General	C	1,224	0.44	0.55	MPAI	TAS-20
Yu (75)	Central	General	C	918	0.69	0.55	MPATS	TAS-20
Yuan (76)	Central	Dissertation	C	870	0.77	0.35	TMD	TAS-20
Yu (77)	Eastern	General	C	1,081	0.69	0.57	MPATS	TAS-20
Hou et al. (78)	Eastern	General	C	1,028	0.70	0.55	MPATS	TAS-20
Zhang (79)	Western	General	C	1,062	0.60	0.39	MPATS	TAS-20

*C, collegestudent; M, middle school student; MPAI, Mobile phone addiction index; SQAPMPU, Self-rating Questionnaire for Adolescent Problematic Mobile Phone Use; CSMPDQ, College students mobile phone dependence questionnaire; MPATS, Mobile Phone Addiction Tendency Scale; NMP-Q, The Nomophobia Questionnaire; SAS-SV, the Smartphone Addiction Scale—Short Version; TMD, The Test of Mobile Phone Dependence; AQCS, Alexithymia Questionnaire of College Students; TAS-20, Toronto alexithymia scale; N, Not reported*.

### Homogeneity Tests and Pooled Analyses

**Figure 2:**
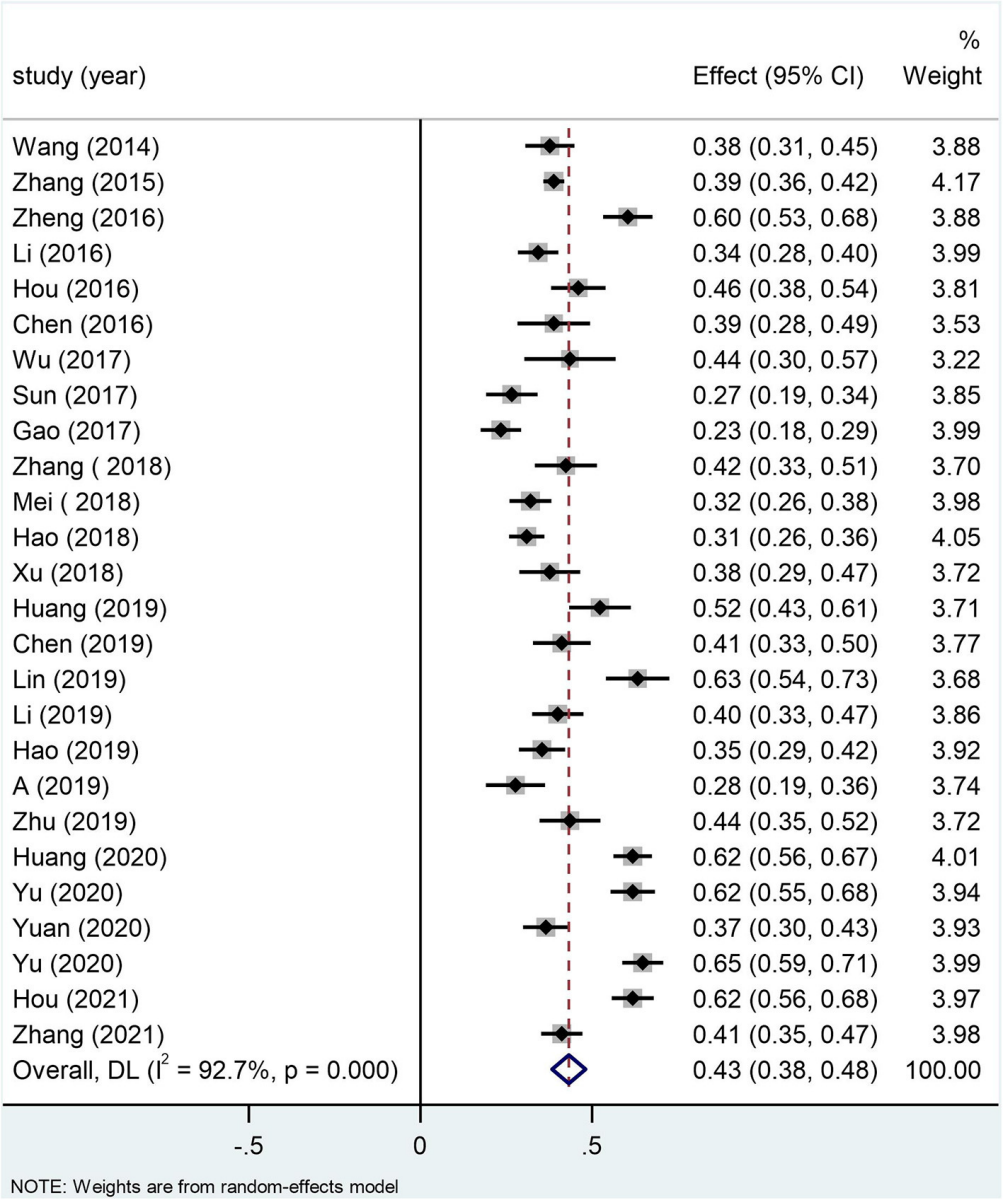
Forest plots for the correlation between alexithymia and mobile phone addiction.

As shown in Table 2, the homogeneity test for 26 independent samples showed substantial heterogeneity among the selected studies (Q-statistic = 343.65; *p* < 0.001; *I*^2^ = 92.7) and likely moderation effects. The random effects model showed a significant correlation of 0.41 (95% CI: 0.37–0.45) between alexithymia and MPA. According to the recommendation of Gignac and Szodorai (53), correlations of 0.10, 0.20, and 0.30 are considered relatively small, typical, and relatively large. There was a relatively large positive correlation between alexithymia and MPA among Chinese students. Moreover, the correlation between alexithymia and MPA was stable, as shown by the *Z*-value of 17.38 and *p* < 0.001 (Figure 2).

**Table 2:** Random-model of the correlation between alexithymia and MPA.

**K**	***N***	**Effect size (r)**	**95% CI for r**	**Homogeneity test**			**Test of null (two tailed)**	
				**Q (r)**	***p***	***I* ^2^**	***Z*-value**	***p***
26	23,687	0.41	[0.37, 0.45]	343.65	0.00	92.7	17.38^***^	<0.001

*** *P < 0.001*.

### Moderator Analysis

A meta-analysis of variance (ANOVA) test was conducted for the moderating effects of key categorical variables: alexithymia measures, MPA measures, publication year, and regional difference. In addition, meta-regression analysis tests for the moderating effects of a key continuous variables: proportion of females.

### Meta-ANOVA

The meta-ANOVA showed that MPA measures, and publication year significantly moderated the relationship between alexithymia and MPA (Table 3).

**Table 3:** Alexithymia and MPA: univariate analysis of variance for moderator variables.

	**Q_**BET**_**	**k**	***N***	**r**	**95% CI for r**	**SE**	**Q_**W**_**	***I* ^2^**
**Alexithymia measures**	0.48							
TAS-20		24	22,385	0.41	[0.36, 0.45]	0.27	342.37^**^	93.3%
ACQS		2	1,002	0.38	[0.33, 0.44]	0.32	0.86^**^	0.00
**MPA measures**	11.56^*^							
MPAI		12	9,991	0.36	[0.31, 0.42]	0.34	117.54^**^	90.6%
MPATS		10	7,813	0.47	[0.41, 0.52]	0.29	83.36^**^	89.2%
Others		4	5,583	0.37	[0.35, 0.39]	0.19	0.92^**^	0.00
**Age**	0.51							
College student		22	20,052	0.40	[0.35, 0.44]	0.28	280.79^**^	92.5%
Middle school student		4	3,335	0.45	[0.31, 0.57]	0.59	62.31^**^	95.2%
**Publication year**	8.66^*^							
2014–2016		6	7,702	0.40	[0.34, 0.46]	0.33	37.19^**^	86.6%
2017–2019		14	9,502	0.36	[0.32, 0.41]	0.30	87.08^**^	85.1%
2020–2021		6	6,183	0.50	[0.42, 0.57]	0.35	74.75^**^	93.3%
**Region**	0.29							
Eastern		9	7,538	0.40	[0.31, 0.49]	0.47	192.37^**^	95.8%
Central		14	14,095	0.41	[0.36, 0.46]	0.29	150.65^**^	91.4%
Western		2	1,534	0.39	[0.35, 0.43b]	0.27	0.05	0.00

* *p < 0.05*,

** *p < 0.001*.

**Figure 3:**
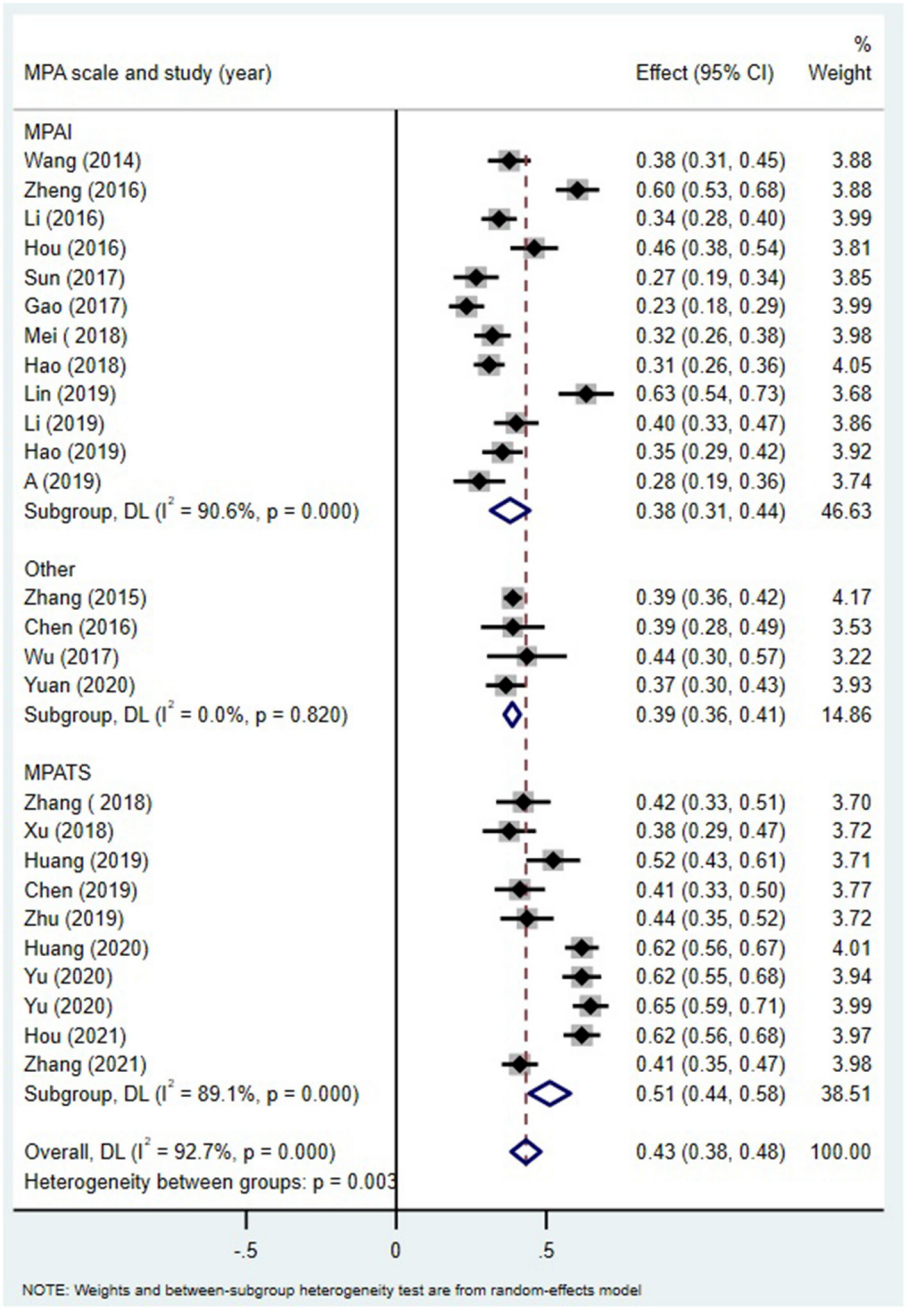
MPA and alexithymia: an analysis of the moderating effects of the MPA measurement tool.

MPA measures significantly moderated the relation between alexithymia and MPA in Chinese students (Q = 11.56, df = 2, *p* < 0.05; Figure 3). This positive correlation coefficient was the largest when using the MPATS (r = 0.47, 95% CI = [0.41, 0.52]), but it was relatively small when MPA was measured with the MPAI (r = 0.36, 95% CI = [0.31, 0.42]) or others (r = 0.37, 95% CI = [0.35, 0.39]).

**Figure 4:**
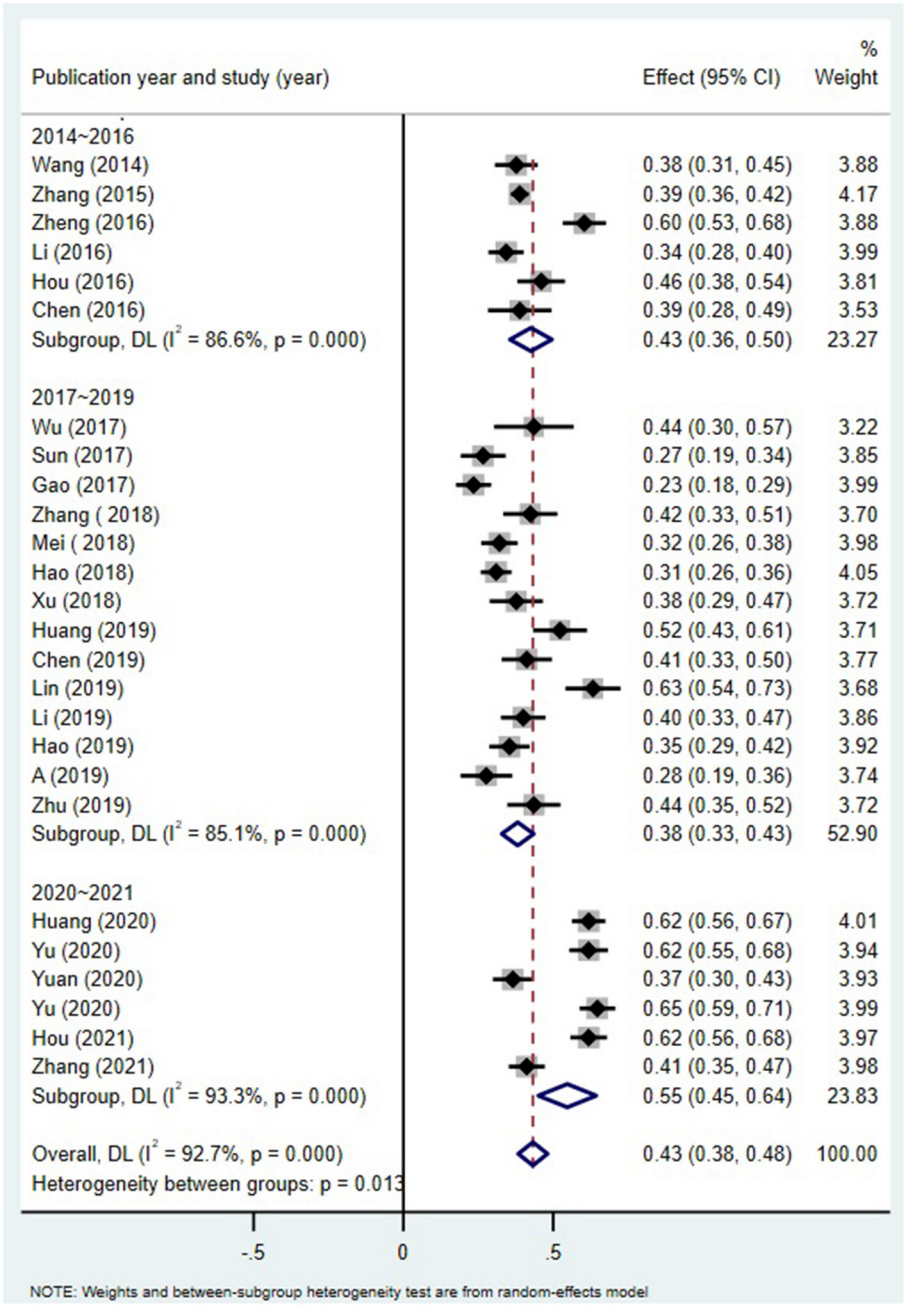
MPA and alexithymia: an analysis of the moderating effects of publication year.

Publication year significantly moderated the relation between alexithymia and MPA (Q = 8.66, df = 2, *p* < 0.05; Figure 4). The correlation is the strongest in 2020–2021 (r = 0.50, 95% CI = [0.42, 0.57]) and smallest in 2017–2019 (r = 0.36, 95% CI = [0.32, 0.41]).

**Figure 5:**
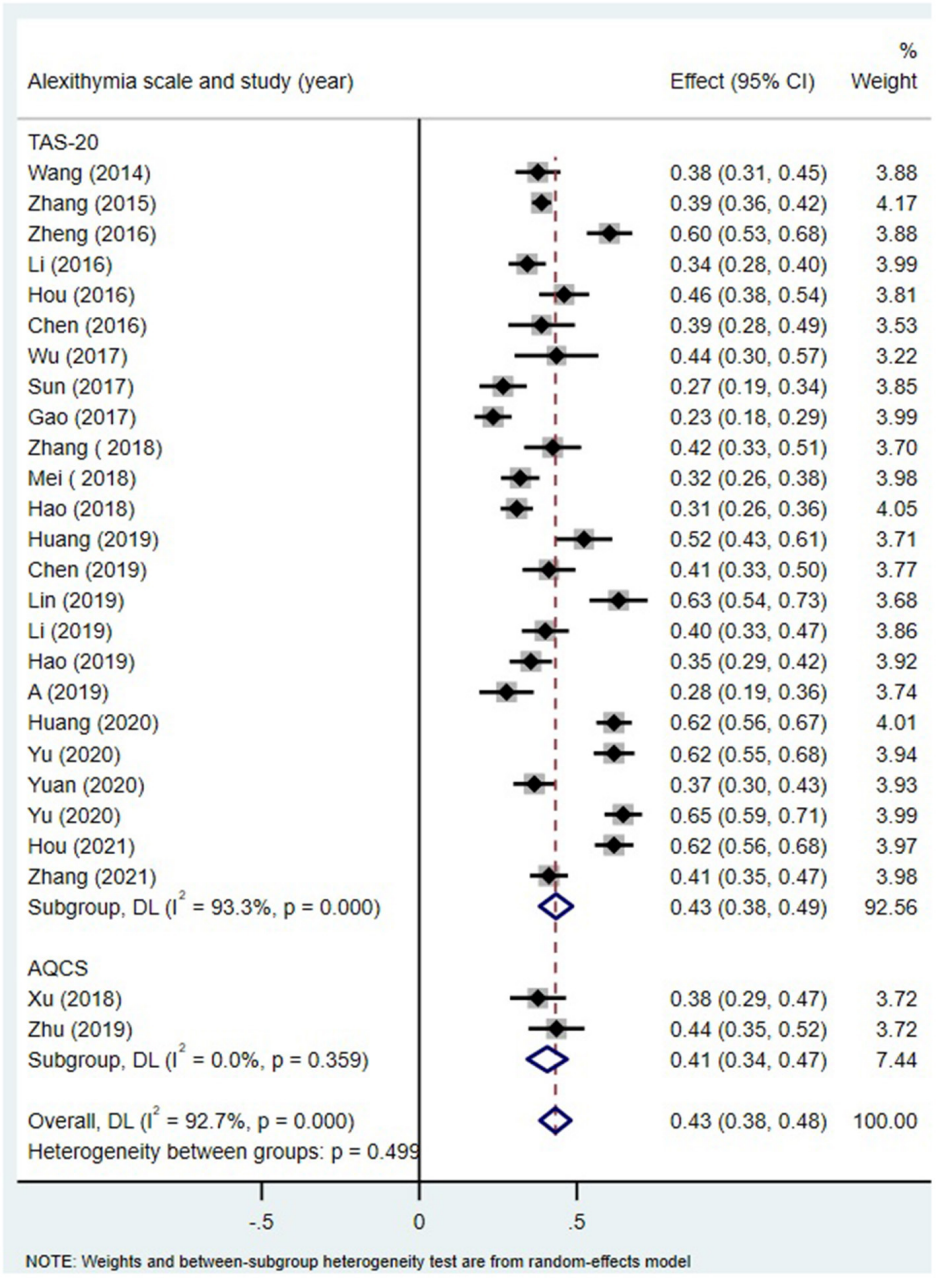
MPA and alexithymia: an analysis of the moderating effects of the alexithymia measurement tool.

**Figure 6:**
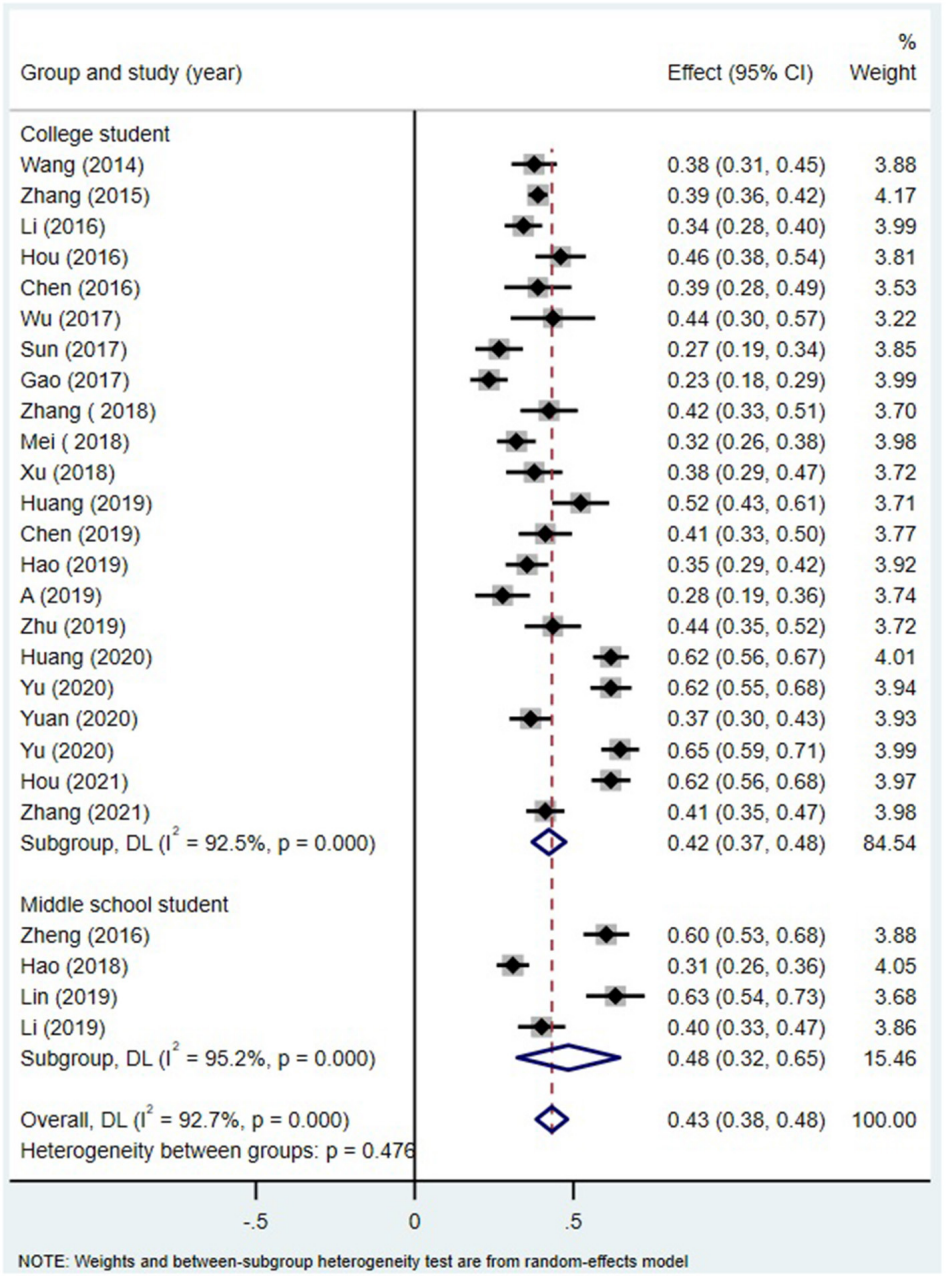
MPA and alexithymia: an analysis of the moderating effects of age group.

**Figure 7:**
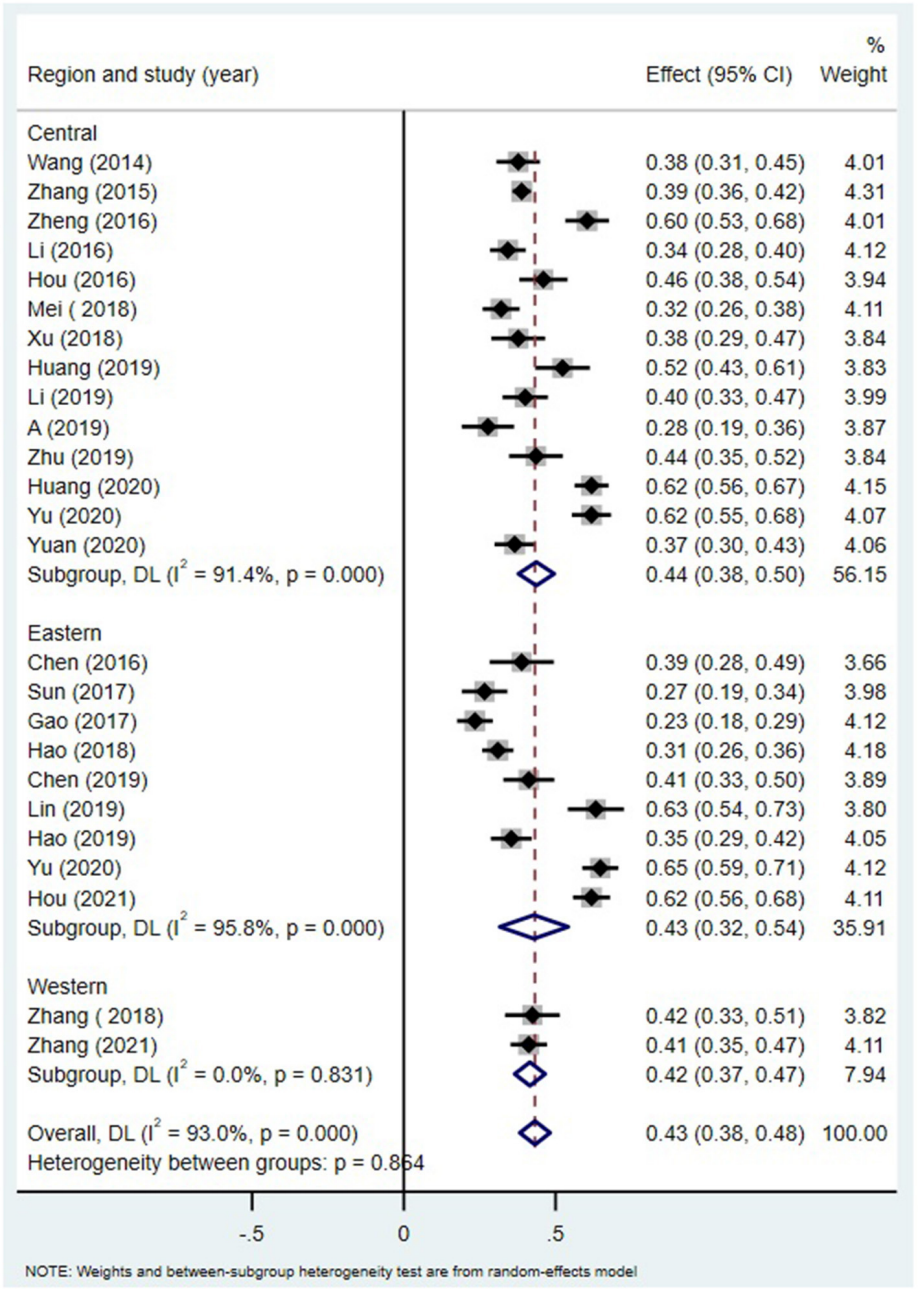
MPA and alexithymia: an analysis of the moderating effects ofregion.

Alexithymia measures (Figure 5), age of the participant group (Figure 6) and region (Figure 7) all did not moderate the correlation between alexithymia and MPA.

### Meta-Regression Analysis

As shown in Table 4, a meta-regression analysis was used to examine whether sex moderated the correlation between alexithymia and MPA among Chinese students. The results show that the link between alexithymia and MPA was not moderated by gender.

**Table 4:** Univariate regression analysis of gender (random-effect model).

***z***	**Coef**.	**SE**	***t***	***P* > |t|**	**95% CI**
Female ratio	0.11	0.17	0.62	0.54	−0.25, 0.46
_cons	0.37	0.09	3.89	0.001	0.17, 0.57

### Sensitivity Analyses

**Figure 8:**
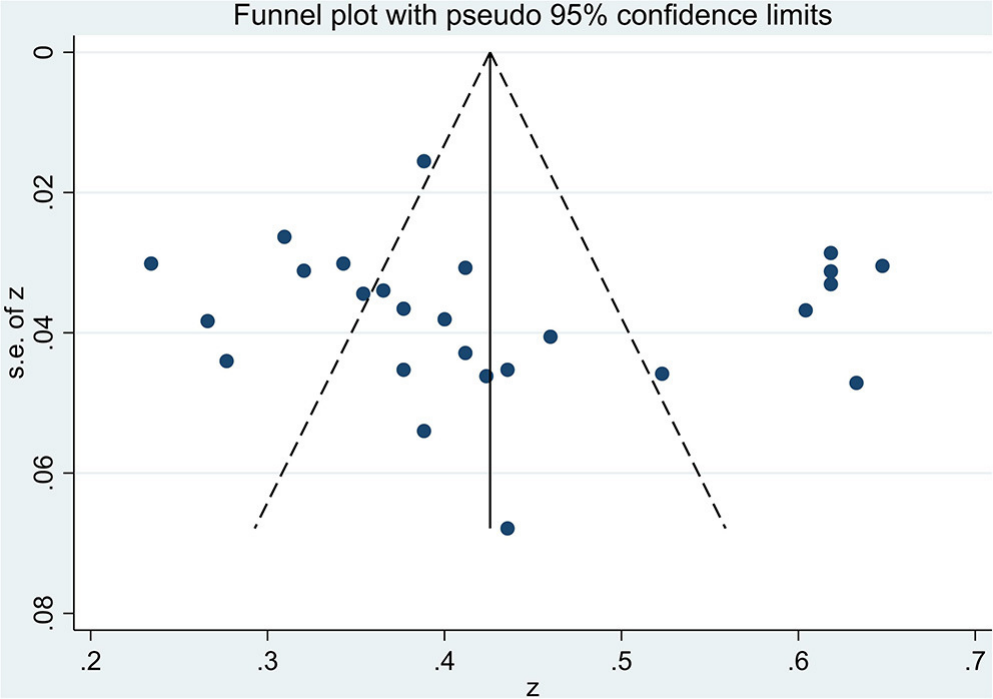
Funnel plot of the 26 studies included in the meta-analysis.

To evaluate the robustness of our findings, sensitivity analyses were performed by sequentially removing one individual study each turn and then recalculating the summary correlation coefficients. Sensitivity analyses for summary correlation coefficients between MPA and alexithymia revealed minor changes, indicating that our results were stable (Figure 8).

### Publication Bias

**Figure 9:**
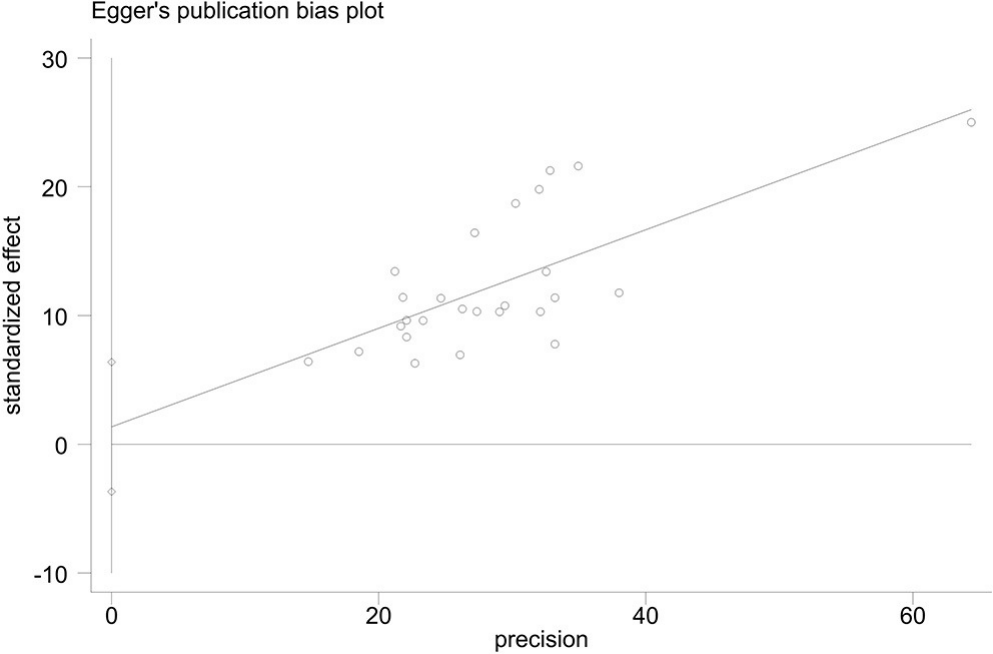
Egger's test of the 26 studies included in the meta-analysis.

**Figure 10:**
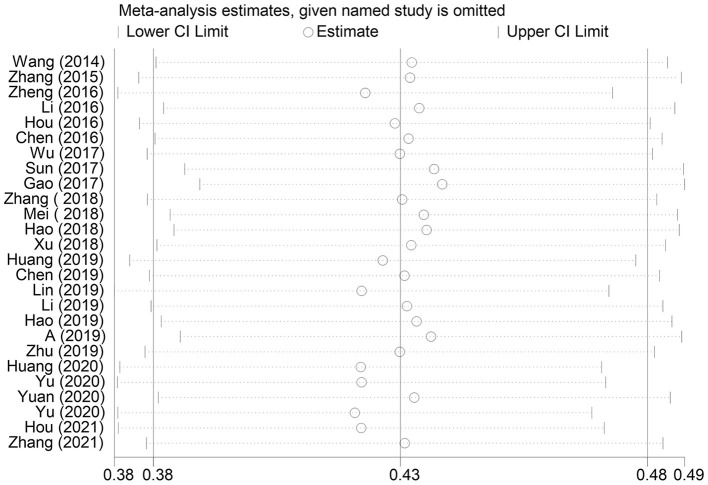
Sensitivity analyses of the 26 studies included in the meta-analysis.

Judging subjectively, it was difficult to determine whether the funnel plots for the summary correlation coefficients between MPA and alexithymia were symmetric (Figure 9). Egger's regression showed no significant bias (t_26_ = 0.56, *p* = 0.58) (Figure 10). The trim-and-fill analysis also revealed no trimming performed and the data did not change (see Supplementary Material 3), indicating that no significant publication bias was detected.

## Discussion

To the best of our knowledge, this was the first meta-analysis exploring the pooled correlation coefficients of MPA with alexithymia among Chinese students. Our aim is to expand the existing knowledge about the relationship between MPA and alexithymia among Chinese students to provide a basis for formulating strategies to promote the physical and mental health development of Chinese students.

### Relation Between Alexithymia and MPA

The meta-analysis results showed that alexithymia had a large positive correlation with MPA among Chinese students (r = 0.41). The cognitive-behavioral theory of pathological internet use holds that psychopathological status and maladjustment are important factors affecting MPA (80). In the process of real interpersonal communication, students with alexithymia often have some problems, such as low sensitivity to understand emotional changes and lack of verbal expression ability, which have a negative impact on their normal interpersonal communication (81). Smartphones have the advantages of easy access and versatility. They can not only help individuals carry out online social communication and establish virtual social relationships to meet their needs of belonging but also help individuals carry out online entertainment and recreation so that the body can become excited and temporarily relieve their inner helplessness and loneliness (82). When they choose to communicate through mobile phones, they can hide their true feelings and avoid getting along with others face to face. In this way, people with alexithymia can show a more comfortable mental state to alleviate discomfort in real communication (36, 81). This also explains that the impact of alexithymia on mobile phone dependence plays a role by making individuals pay too much attention to maintaining social relations and social comfort, because individuals with alexithymia prefer to obtain social support and comfort through mobile phones, and it is easier to regard mobile phones as the best choice instead of face-to-face communication (74). In addition, in a study with Chinese students as samples, researchers found that alexithymia not only has a direct positive impact on MPA but also has an indirect impact on MPA through depression, anxiety or stress (22). The same result was found by Lyvers et al. (83). The reason may be that alexithymia patients have defects in emotional cognitive processing and empathy (84), bad coping styles (85) and social support in understanding (86), which usually cannot deal with stress situations well, aggravating negative emotions, such as depression and anxiety. Another study on Chinese students' MPA and alexithymia also found that alexithymia can also affect MPA through self-esteem (69). Self-esteem is an individual's evaluation of self and an important psychological resource in the structure of self-system (87). Alexithymia leads to inconsistency between individual implicit self-esteem and explicit self-esteem, prevents individual emotions from being moderated and adjusted, and induces individual MPA behavior (88).

On the other hand, MPA may also affect the level of alexithymia. According to cognitive-behavioral theory, individuals' cognitions and emotions could not only affect their behaviors but also be influenced by their own behaviors (89). Students with a high degree of MPA tend to neglect offline social communication, which reduces the time and opportunities for offline communication with friends and family and has a negative impact on the establishment of a social emotional support system. When students transfer from the internet world to the real world, they may feel more social alienation, resulting in social isolation and unwillingness to communicate with society (43, 90). Therefore, the level of alexithymia may increase.

### Moderation Effects

#### Moderating Role of Alexithymia Measures

The analysis showed that although the research using the TAS-20 had a higher correlation between alexithymia and MPA than those using the AQCS, the difference was not significant (Q = 0.48, *p* = 0.49). This indicates that alexithymia measures did not moderate the correlation between alexithymia and MPA among Chinese students. On the one hand, this may be due to the stability of the correlation between alexithymia and MPA across measures, and on the other hand, it may be related to the lack of studies that used the AQCS (only two studies). Therefore, the results of this study cannot fully reflect the relationship between alexithymia and MPA under different alexithymia measurement tools. The results of this study still need to be confirmed by further studies.

#### Moderating Role of MPA Measures

The measure of MPA moderates the large positive correlation between alexithymia and MPA. This positive correlation is smaller when MPA is measured with MPAI and other (r = 0.37, 0.36) than with MAPTS measures (0.47). Similar results were also reported in a meta-analysis of the relationship between internet addiction and social support (91). This raises the possibility that a lack of acculturation to the mainland Chinese environment may be responsible for the relatively small positive correlation results. Since MAPTS were developed for the Chinese population, they are unlikely to have this problem. In addition, because the MPA measurement tools except MPAI and MPATS are classified as other measures in this study, whether the relationship between alexithymia and MPA is moderated by other less used MPA measures still needs further exploration.

#### Moderating Role of Age and Gender

Age did not moderate the positive relation between alexithymia and MPA among Chinese students. On the one hand, this may be because college students and middle school students both live in similar cultural atmospheres and social environments, so external environmental factors have the same influence on them, and there is little difference in social support they have received. On the other hand, the age and psychological development level of college students and middle school students are relatively close (92). During this period, they were far away from their parents and began to face study and life alone. To avoid loneliness, they need to obtain more social support. If they cannot obtain satisfaction and emotional vents in real life, they can use mobile phones to meet this demand, which makes them prone to be dependent on mobile phones.

Meta-regression showed that gender had no significant moderating effect on alexithymia and MPA among Chinese students. This suggests that the relationship between alexithymia and MPA may be stable across genders. Although individuals of different genders may have different preferences for the specific content of mobile phone use, boys may prefer gaming apps, while girls may prefer social apps, and there may be no significant gender difference in the overall degree of mobile phone use (93, 94). A meta-analysis similar to this study also found that the relationship between MPA and anxiety/depression was not moderated by gender (95). This suggests that it may be more common to use a phone to defuse negative feelings when Chinese students' alexithymia cannot be alleviated.

#### Moderating Role of Regions

The relationship between alexithymia and MPA was not moderated by region. This shows that MPA may be a common problem among students in China, and there is no regional difference. This may be related to the decline in the price of smartphones (96, 97) and the rapid development of mobile internet in China, which has intensified the use of mobile phones among young people. Mobile phones have become an integral part of their lives (98).

In addition, there are only two studies in the western region, which may have a certain impact on the test of moderating variables. Future studies can further expand the number of studies to further test whether the region plays a moderator in the relationship between alexithymia and mobile phone addiction.

#### Moderating Role of Publication Year

Publication year moderated the positive correlation between alexithymia and MPA, and the results showed that the correlation was generally enhanced with the development of time. This is consistent with the results of a meta-analysis on the relationship between social support and mobile phone dependence of Chinese college students (99). The reason may be that with the popularity of mobile internet, mobile phones play an increasingly important role in Chinese student's lives: they use mobile phones for a longer time, and the frequency of using mobile phones for communication and entertainment is also increasing. Studies have shown that with the popularity of mobile phones and their use years getting longer, the problem of MPA is more likely to occur (56). Additionally, a high level of alexithymia is related to negative social support and maladjustment (100). If individuals have a higher level of alexithymia, MPA is more likely to occur. Therefore, the correlation between alexithymia and MPA is increasing over time. This also reminds us from another point of view that on the one hand, we need to pay attention to the personality traits of teenagers and provide personality quality education for students; on the other hand, we need to constantly improve the social support system of students. However, the studies in this meta-analysis were published in the past 8 years, and the time span is small. Additionally, the number of studies published from 2014–2016 and 2020–2021 is small, which may have s limited the research results.

## Limitations and Prospects

This study has the following limitations. First, it should be noted that there is no consensus on the concept of MPA at present, so the literature included in this study also includes research on problematic mobile phone use, mobile phone dependence and mobile phone overuse. Second, this study only focuses on the relationship between alexithymia and general mobile phone addiction. Future studies can also analyze the relationship between alexithymia and specific social network addiction or online game addiction. Third, the studies in this study were all cross-sectional studies. In the future, longitudinal research design can be added to further clarify the causal role of alexithymia and mobile phone addiction. Finally, this study only focused on the simple correlation between alexithymia and MPA. Future research can further focus on the psychological variables (e.g., introverted personality, self-disclosure, etc.) that directly affect the relationship between alexithymia and mobile phone addiction in Chinese students, so as to provide a clearer idea for future research on mental health interventions.

## Conclusion

There was a positive correlation between alexithymia and MPA among Chinese students. Students with higher alexithymia levels are more dependent on mobile phones, and vice versa. Furthermore, the relationship was moderated by mobile phone addiction measurement tool and year of publication, with studies using the MPATS having higher correlation coefficients than those using the MPAI or other measurement tools. Studies published in 2020–2021 yielded higher correlations than those published in 2014–2016 and 2017–2019. However, the relationship was not moderated by gender, region, or the measures of alexithymia. Longitudinal studies should be conducted in the future to further reveal the relationship between alexithymia and mobile phone addiction in Chinese students over time.

## Author Contributions

HH and XW: study design, critical revision of the manuscript, and drafting of the manuscript. GL, YD, CC, and HH: analysis and interpretation of data. All authors approval of the final version for submission.

## Funding

This research was Sponsored by Graduate Education Reform and Quality Improvement Project of Henan Province (Grant Number: YJS2021AL074), Graduate Education Innovation and Quality Improvement Project of Henan University (Grant Number: SYL19060141), and Planning and Decision Consultation Project of Henan Province (Grant Number: 2018JC38).

## Conflict of Interest

The authors declare that the research was conducted in the absence of any commercial or financial relationships that could be construed as a potential conflict of interest.

## Publisher's Note

All claims expressed in this article are solely those of the authors and do not necessarily represent those of their affiliated organizations, or those of the publisher, the editors and the reviewers. Any product that may be evaluated in this article, or claim that may be made by its manufacturer, is not guaranteed or endorsed by the publisher.
