# Supplementary material for: The Relationship Between Alexithymia and Mobile Phone Addiction Among Mainland Chinese Students: A Meta-Analysis
Source: Front Psychiatry. 2022 Feb 10;13:754542. doi: 10.3389/fpsyt.2022.754542 (PMC8866180; doi:10.3389/fpsyt.2022.754542)

**Figure 1 Calculation results before applying the trim and fill method**


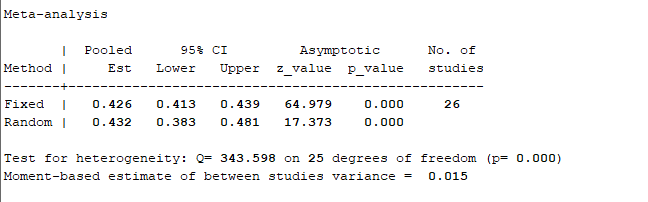


**Figure 2 Calculation results after applying the trim and fill method**


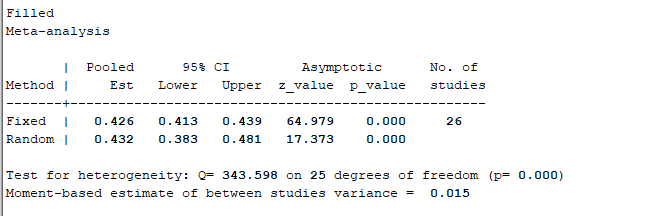


**Figure 3 Funnel plot after applying the trim and fill method**


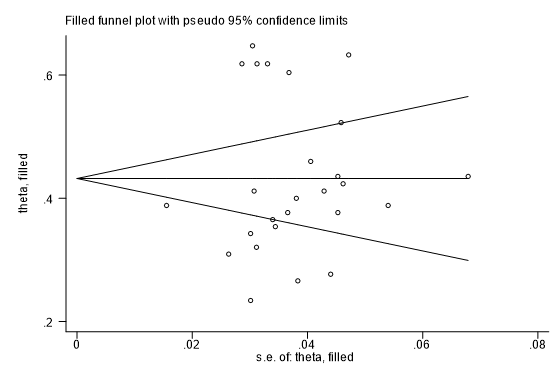

Supplement: Supplementary file 1 [file Data_Sheet_1.docx]
