# Supplementary material for: The Relationship Between Alexithymia and Mobile Phone Addiction Among Mainland Chinese Students: A Meta-Analysis
Source: Front Psychiatry. 2022 Feb 10;13:754542. doi: 10.3389/fpsyt.2022.754542 (PMC8866180; doi:10.3389/fpsyt.2022.754542)
Supplement: Supplementary file 2 [file Table_1.DOCX]

**SEARCH STRATEGY IN PUBMED DATABASE**

| Search | Query | Items found | Time |
| --- | --- | --- | --- |
| #7 | Search: ((((((((((((cell phone[Title/Abstract]) OR (cell phones[Title/Abstract])) OR (cellular phone[Title/Abstract])) OR (cellular phones[Title/Abstract])) OR (cellular telephone[Title/Abstract])) OR (cellular telephones[Title/Abstract])) OR (mobile devices[Title/Abstract])) OR (mobile phone[Title/Abstract])) OR (smart phone[Title/Abstract])) OR (smartphone[Title/Abstract])) AND ((((((((addiction[Title/Abstract]) OR (dependence[Title/Abstract])) OR (dependency[Title/Abstract])) OR (abuse[Title/Abstract])) OR (addicted to[Title/Abstract])) OR (overuse[Title/Abstract])) OR (problem use[Title/Abstract])) OR (compensatory use[Title/Abstract]))) OR (((((((((((((((problematic smartphone use[Title/Abstract]) OR (problematic smart phone use[Title/Abstract])) OR (problematic mobile phone use[Title/Abstract])) OR (problematic cell phone use[Title/Abstract])) OR (problematic cell phone use[Title/Abstract])) OR (Nomophobia[Title/Abstract])) OR (smartphone zombies[Title/Abstract])) OR (Phubbing[Title/Abstract])) OR (fear of missing out[Title/Abstract])) OR (FoMO[Title/Abstract])) OR (smartphone separation anxiety[Title/Abstract])) OR (smartphone use disorder[Title/Abstract])) OR (compulsive mobile phone use[Title/Abstract])) OR (fear of being without a mobile phone[Title/Abstract])) OR (fear of being without a smartphone[Title/Abstract]))) AND (((((((((Affective Symptom[Title/Abstract]) OR (Symptom, Affective[Title/Abstract])) OR (Symptoms, Affective[Title/Abstract])) OR (Alexithymia[Title/Abstract])) OR (Alexithymias[Title/Abstract])) OR (Emotional Disturbances[Title/Abstract])) OR (Disturbance, Emotional[Title/Abstract])) OR (Disturbances, Emotional[Title/Abstract])) OR (Emotional Disturbance[Title/Abstract])) | 7 | 08:49:54 |
| #6 | Search: ((((((((Affective Symptom[Title/Abstract]) OR (Symptom, Affective[Title/Abstract])) OR (Symptoms, Affective[Title/Abstract])) OR (Alexithymia[Title/Abstract])) OR (Alexithymias[Title/Abstract])) OR (Emotional Disturbances[Title/Abstract])) OR (Disturbance, Emotional[Title/Abstract])) OR (Disturbances, Emotional[Title/Abstract])) OR (Emotional Disturbance[Title/Abstract]) | 5327 | 08:47:59 |
| #5 | Search: (((((((((((cell phone[Title/Abstract]) OR (cell phones[Title/Abstract])) OR (cellular phone[Title/Abstract])) OR (cellular phones[Title/Abstract])) OR (cellular telephone[Title/Abstract])) OR (cellular telephones[Title/Abstract])) OR (mobile devices[Title/Abstract])) OR (mobile phone[Title/Abstract])) OR (smart phone[Title/Abstract])) OR (smartphone[Title/Abstract])) AND ((((((((addiction[Title/Abstract]) OR (dependence[Title/Abstract])) OR (dependency[Title/Abstract])) OR (abuse[Title/Abstract])) OR (addicted to[Title/Abstract])) OR (overuse[Title/Abstract])) OR (problem use[Title/Abstract])) OR (compensatory use[Title/Abstract]))) OR (((((((((((((((problematic smartphone use[Title/Abstract]) OR (problematic smart phone use[Title/Abstract])) OR (problematic mobile phone use[Title/Abstract])) OR (problematic cell phone use[Title/Abstract])) OR (problematic cell phone use[Title/Abstract])) OR (Nomophobia[Title/Abstract])) OR (smartphone zombies[Title/Abstract])) OR (Phubbing[Title/Abstract])) OR (fear of missing out[Title/Abstract])) OR (FoMO[Title/Abstract])) OR (smartphone separation anxiety[Title/Abstract])) OR (smartphone use disorder[Title/Abstract])) OR (compulsive mobile phone use[Title/Abstract])) OR (fear of being without a mobile phone[Title/Abstract])) OR (fear of being without a smartphone[Title/Abstract])) | 1,378 | 08:47:59 |
| #4 | Search: ((((((((((((((problematic smartphone use[Title/Abstract]) OR (problematic smart phone use[Title/Abstract])) OR (problematic mobile phone use[Title/Abstract])) OR (problematic cell phone use[Title/Abstract])) OR (problematic cell phone use[Title/Abstract])) OR (Nomophobia[Title/Abstract])) OR (smartphone zombies[Title/Abstract])) OR (Phubbing[Title/Abstract])) OR (fear of missing out[Title/Abstract])) OR (FoMO[Title/Abstract])) OR (smartphone separation anxiety[Title/Abstract])) OR (smartphone use disorder[Title/Abstract])) OR (compulsive mobile phone use[Title/Abstract])) OR (fear of being without a mobile phone[Title/Abstract])) OR (fear of being without a smartphone[Title/Abstract]) | 431 | 08:46:54 |
| #3 | Search: ((((((((((cell phone[Title/Abstract]) OR (cell phones[Title/Abstract])) OR (cellular phone[Title/Abstract])) OR (cellular phones[Title/Abstract])) OR (cellular telephone[Title/Abstract])) OR (cellular telephones[Title/Abstract])) OR (mobile devices[Title/Abstract])) OR (mobile phone[Title/Abstract])) OR (smart phone[Title/Abstract])) OR (smartphone[Title/Abstract])) AND ((((((((addiction[Title/Abstract]) OR (dependence[Title/Abstract])) OR (dependency[Title/Abstract])) OR (abuse[Title/Abstract])) OR (addicted to[Title/Abstract])) OR (overuse[Title/Abstract])) OR (problem use[Title/Abstract])) OR (compensatory use[Title/Abstract])) | 1,378 | 08:38:30 |
| #2 | Search: (((((((addiction[Title/Abstract]) OR (dependence[Title/Abstract])) OR (dependency[Title/Abstract])) OR (abuse[Title/Abstract])) OR (addicted to[Title/Abstract])) OR (overuse[Title/Abstract])) OR (problem use[Title/Abstract])) OR (compensatory use[Title/Abstract]) | 439,372 | 08:38:30 |
| #1 | Search: (((((((((cell phone[Title/Abstract]) OR (cell phones[Title/Abstract])) OR (cellular phone[Title/Abstract])) OR (cellular phones[Title/Abstract])) OR (cellular telephone[Title/Abstract])) OR (cellular telephones[Title/Abstract])) OR (mobile devices[Title/Abstract])) OR (mobile phone[Title/Abstract])) OR (smart phone[Title/Abstract])) OR (smartphone[Title/Abstract]) | 24,557 | 08:36:06 |
