# Supplementary material for: The Relationship Between Alexithymia and Mobile Phone Addiction Among Mainland Chinese Students: A Meta-Analysis
Source: Front Psychiatry. 2022 Feb 10;13:754542. doi: 10.3389/fpsyt.2022.754542 (PMC8866180; doi:10.3389/fpsyt.2022.754542)
Supplement: Supplementary file 3 [file Table_2.DOCX]

| Study | Year | ① | ② | ③ | ④ | ⑤ | ⑥ | ⑦ | ⑧ | ⑨ | ⑩ | ⑪ |
| --- | --- | --- | --- | --- | --- | --- | --- | --- | --- | --- | --- | --- |
| Wang | 2014 | Y | Y | Y | Y | U | N | N | N | N | Y | N |
| Zhang | 2015 | Y | Y | Y | Y | U | N | Y | N | N | Y | N |
| Zheng | 2016 | Y | Y | Y | Y | U | N | N | N | N | Y | N |
| Li | 2016 | Y | Y | Y | Y | U | Y | Y | Y | N | Y | N |
| Hou | 2016 | Y | Y | Y | Y | U | Y | N | N | N | Y | N |
| Chen | 2016 | Y | Y | Y | Y | U | Y | N | N | N | Y | N |
| Wu | 2017 | Y | Y | Y | Y | U | N | N | N | N | Y | N |
| Sun | 2017 | Y | Y | Y | Y | U | N | N | N | N | Y | N |
| Gao | 2017 | Y | Y | Y | Y | U | Y | Y | Y | N | Y | N |
| Zhang | 2018 | Y | Y | Y | Y | U | Y | N | Y | N | N | N |
| Mei | 2018 | Y | Y | Y | Y | U | Y | Y | Y | N | Y | N |
| Hao | 2018 | Y | Y | Y | Y | U | Y | Y | Y | N | Y | N |
| Xu | 2018 | Y | Y | Y | Y | U | N | N | N | N | Y | N |
| Huang | 2019 | Y | Y | Y | Y | U | N | Y | N | N | Y | N |
| Chen | 2019 | Y | Y | Y | Y | U | Y | N | Y | N | Y | N |
| Lin | 2019 | Y | Y | Y | Y | U | Y | N | N | N | Y | N |
| Li | 2019 | Y | Y | Y | Y | U | Y | N | N | N | Y | N |
| Hao | 2019 | Y | Y | Y | Y | U | N | N | N | N | Y | N |
| A | 2019 | Y | Y | Y | Y | U | N | N | N | N | Y | N |
| Zhu | 2019 | Y | Y | Y | Y | U | Y | Y | N | N | Y | N |
| Huang | 2020 | Y | Y | Y | Y | U | Y | Y | Y | N | N | N |
| Yu | 2020 | Y | Y | Y | Y | U | Y | N | Y | N | Y | N |
| Yuan | 2020 | Y | Y | Y | Y | U | Y | Y | Y | N | Y | N |
| Yu | 2020 | Y | Y | Y | Y | U | N | N | N | N | Y | N |
| Hou | 2021 | Y | Y | Y | Y | U | N | Y | N | N | Y | N |
| Zhang | 2021 | Y | Y | Y | Y | U | Y | Y | Y | N | Y | N |

Y:Yes;N:No;U:Uclear; ①Define the source of information (survey, record review)；②List inclusion and exclusion criteria for exposed and unexposed subjects(cases and controls) or refer to previous publications; ③Indicate time period used for identifying patients; ④indicate whether or not subjects were consecutive if not population-based; ⑤indicate if evaluators of subjective components of study were masked to other aspects of the status of the participants; ⑥Describe any assessments undertaken for quality assurance purposes(e.g., test/retest of primary outcome measurements); ⑦Explain any patient exclusions from analysis; ⑧Describe how confounding was assessed and/or controlled; ⑨If applicable, explain how missing data were handled in the analysis; ⑩Summarize patient response rates and completeness of data collection; ⑪Clarify what follow-up, was expected and percentage of patients for which incomplete data or follow-up was obtained.
